# Traumatic Ulcer Caused by Supragingival Calculus: A Case Report

**DOI:** 10.7759/cureus.116296

**Published:** 2026-09-15

**Authors:** Ramachandra Reddy Gowda Venkatesha, Karthik Rajaram Mohan, Saramma Mathew Fenn

**Affiliations:** 1 Oral Medicine and Radiology, Vinayaka Mission's Sankarachariyar Dental College, Vinayaka Mission's Research Foundation (DU), Salem, IND

**Keywords:** lower lip, oral prophylaxis, oral ulcer, traumatic ulcer, ultrasonic scaling

## Abstract

Traumatic ulcer is usually caused by trauma in the form of accidental lip or cheek biting habit, sharp carious tooth, attrited teeth with sharp cusps, or clasps of ill-fitting removable partial dentures. The ulcer clinically appears solitary with sloping edges and sometimes surrounded by a white border due to hyperkeratosis caused by friction, called frictional keratosis. An unusual case of traumatic ulcer in a 36-year-old male caused by a sharp supragingival calculus due to poor oral hygiene and its treatment was discussed in this report.

## Introduction

A traumatic ulcer, with distinctive histopathological characteristics, is an ulcer of the oral mucosa usually caused by trauma. Two of the most prevalent reasons include accidental injuries from biting and malpositioned sharp carious teeth [1]. Self-inflicted injuries resulting from nocturnal lip biting or clenching of the tongue, thermal and electrical injuries, heated meals or beverages, and even factitial injuries such as Munchausen syndrome of proxy produces a similar result [1].

An oral traumatic ulcer is likely underreported, yet it is regarded as less prevalent than an aphthous ulcer. An erythematous halo usually surrounds aphthous ulcers. Traumatic ulcers are more common along the lateral border of the tongue or buccal mucosa along the line of teeth occlusion. Patients with acute traumatic ulcers in their oral cavity usually complain of pain, and fibro purulent membranes often cover such ulcers.The border of induration and hyperkeratosis may be mistaken for squamous cell cancer [1].

An extremely thick fibrinopurulent membrane or exudate at the microscopic level covers an acute traumatic ulcer. The epithelium directly next to the ulcer will frequently exhibit pseudoepitheliomatous hyperplasia, whilst the ulcer bed includes granulation tissue. Granular tissue also contains endothelial proliferation and extensive infiltration of inflammatory cells, e.g. histiocytes, lymphocytes, neutrophils, eosinophils, or even plasma cells [1].

The inflammation may spread to the muscular bundles beneath. The muscle bundle may be atrophic, and the cytoplasm is diminished. Anomaly histiocytes may also be seen on rare occasions. Mitotic figures are typically readily identifiable, whereas necrosis is limited to electrical or thermal burn cases [2].

Dental materials such as sodium hypochlorite as a root canal irrigant and formocresol in pulp medicament can induce traumatic chemical oral ulceration in the oral cavity. Abused drugs such as cocaine, amphetamine, and methylenedioxymethamphetamine (MDMA) also induce traumatic ulcers in the oral cavity [2].

The prevalence of traumatic oral ulcers was seen to be 0.78% in a tertiary care hospital in central India [3]. In traumatic ulcerative granuloma with stromal eosinophilia (TUGSE), there is a possibility of CD30-positive cells, and there have been reports of monoclonal recombinations, although their significance has not yet been determined [4].

## Case presentation

A 36-year-old male presented with a chief complaint of soreness on his lip. History revealed that he used a neem stick for cleaning his teeth. Extraoral examination revealed no lymphadenopathy. The patient had no systemic illness such as diabetes or hypertension. The patient had no harmful oral habits such as smoking or chewing tobacco. Hard tissue examination revealed a sharp supragingival calculus on the mandibular anterior teeth. Intraoral examination revealed an oval-shaped ulcer measuring about 1.5 x 1 cm on the mandibular labial mucosa concerning a sharp supragingival calculus deposit about the left mandibular central incisor (Figure 1). The floor showed a pinkish granulation tissue, and the edges of the ulcer appeared everted. A whitish area surrounded the ulcer. The ulcer was tender on palpation, firm in consistency, and base-non-indurated. Based on the history and clinical findings, a provisional diagnosis of traumatic ulcer was made.

**Figure 1:**
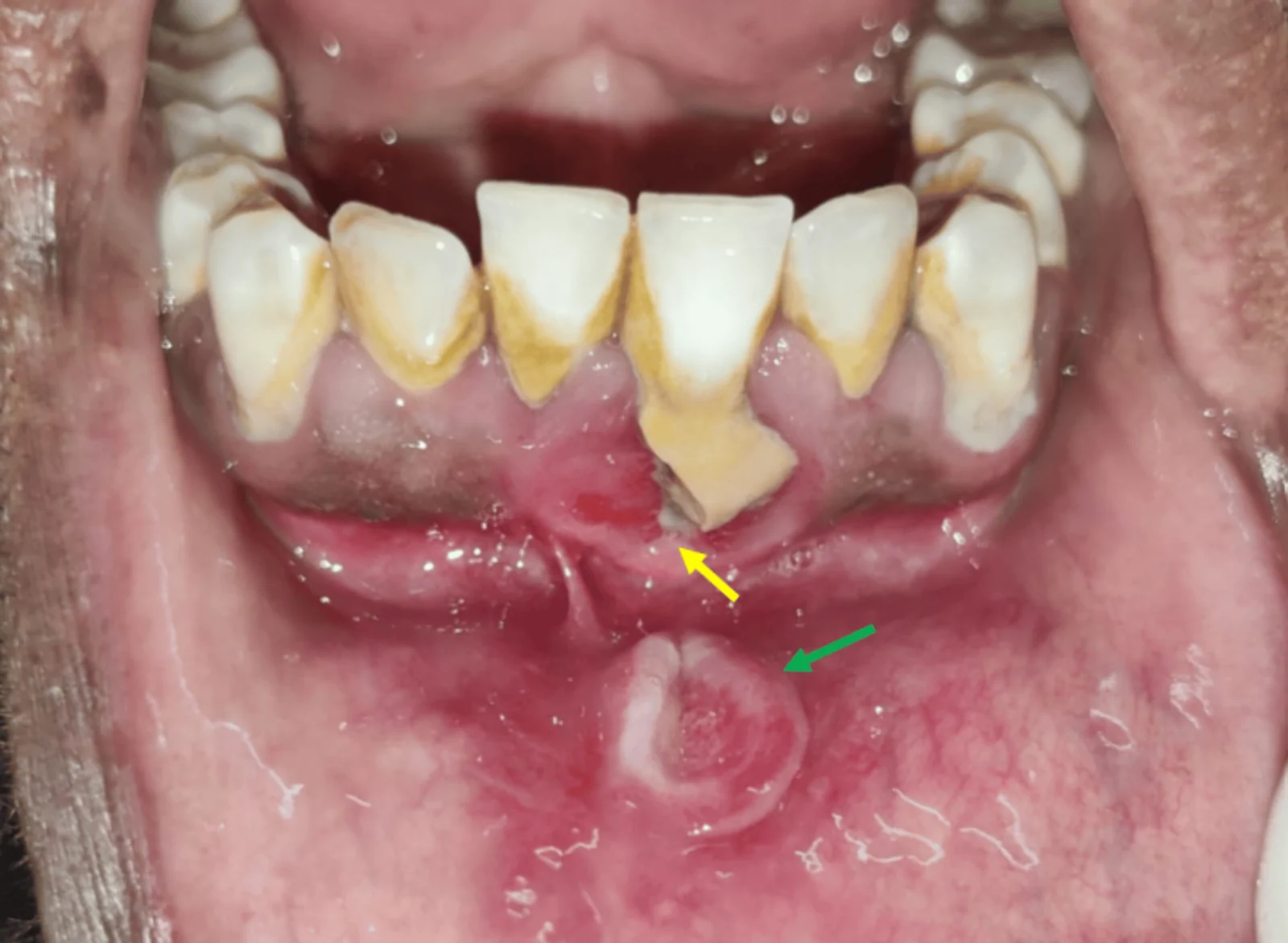
Intraoral examination revealed an ulcer measuring 1.5 x 1 cm on the lip (green arrow) related to sharp supragingival calculus in 31 region (yellow arrow).

Ultrasonic scaling was done, and sharp calculus was removed. A week post-treatment follow-up intraoral clinical photograph revealed a significant reduction in the size of the ulcer and satisfactory healing (Figure 2).

**Figure 2:**
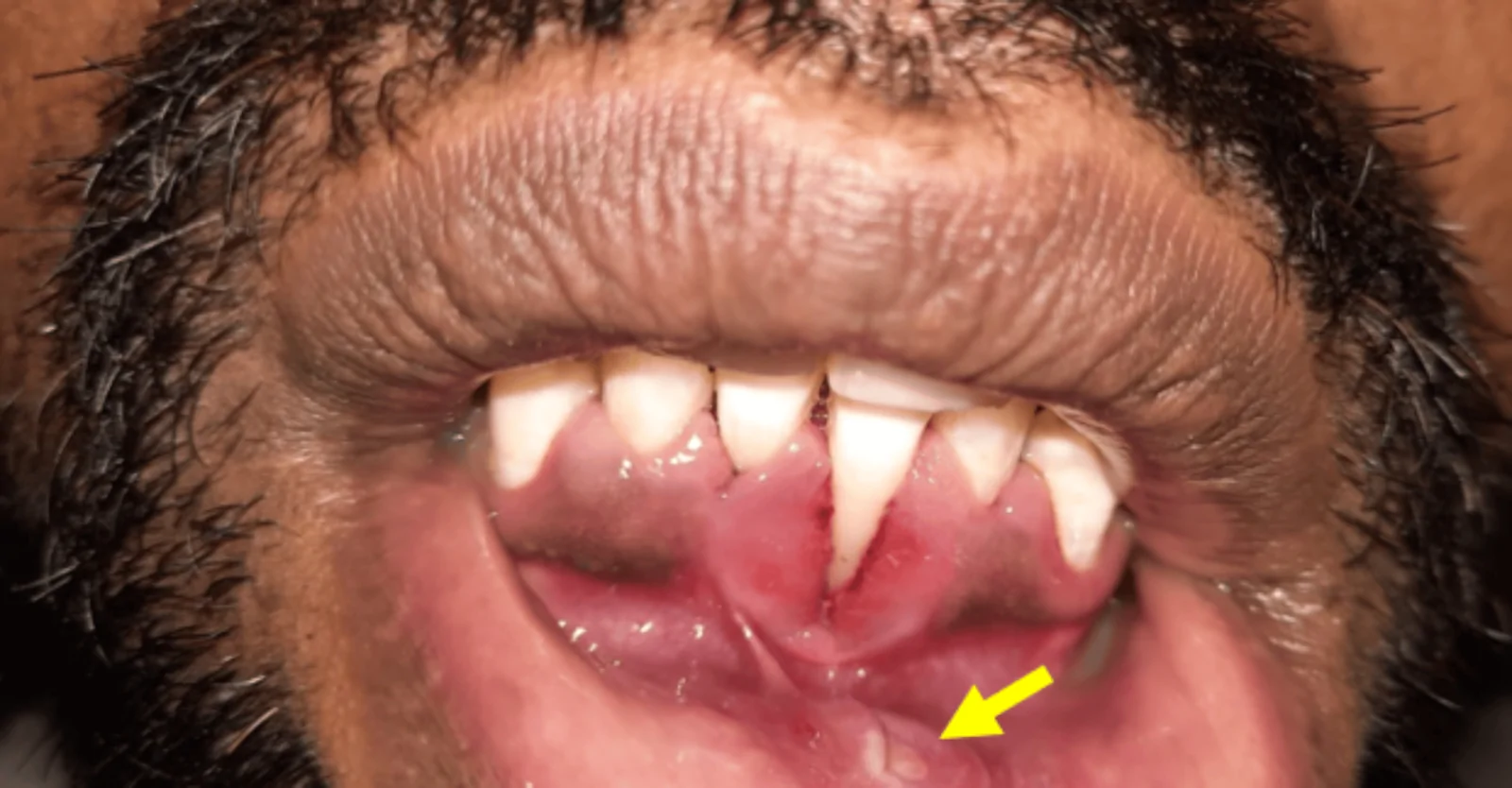
Supragingival calculus was removed by ultrasonic scaling. The healing of the ulcer was satisfactory with reduction in size.

The patient was also educated about proper brushing techniques to avoid the recurrence of the supragingival calculus, which results in traumatic ulcer.

Clinical significance

The case describes an acute solitary traumatic ulcer caused by a sharp supragingival calculus and the need for the importance of good oral hygiene.

Limitation 

Biopsy and special immunohistochemical staining were not done to establish the final diagnosis in this case, as the ulcer showed marked healing after removing the local irritation (supragingival calculus deposit). In this case, further long-term treatment follow-ups in months or years were not done. The absence of an immunohistochemical staining method lacks an evidence-based treatment approach.

## Discussion

Traumatic ulcers are more common in the oral cavity. They usually occur in solitary but are present in acute or chronic forms. In clinical terms, they occur most commonly in the lateral border of the tongue, presenting as a persistent, indurated ulcer with raised or rolled borders. The acute traumatic ulcer is characteristically painful and has a pink floor. The acute traumatic ulcer can also have an everted margin and mimic a carcinomatous ulcer. If the cause is eliminated, it may take 7-10 days for these ulcers to heal [1].

Chronic oral ulcers persist over two weeks. Such ulcers exhibit little to no pain and have elevated, white, non-scrapable keratotic margins due to chronic friction. On palpation, chronic traumatic ulcers are firm due to the infiltration of inflammatory cells of chronic origin, such as lymphocytes, followed by fibrosis [1]. The classification of solitary ulcers is shown in Figure 3.

**Figure 3:**
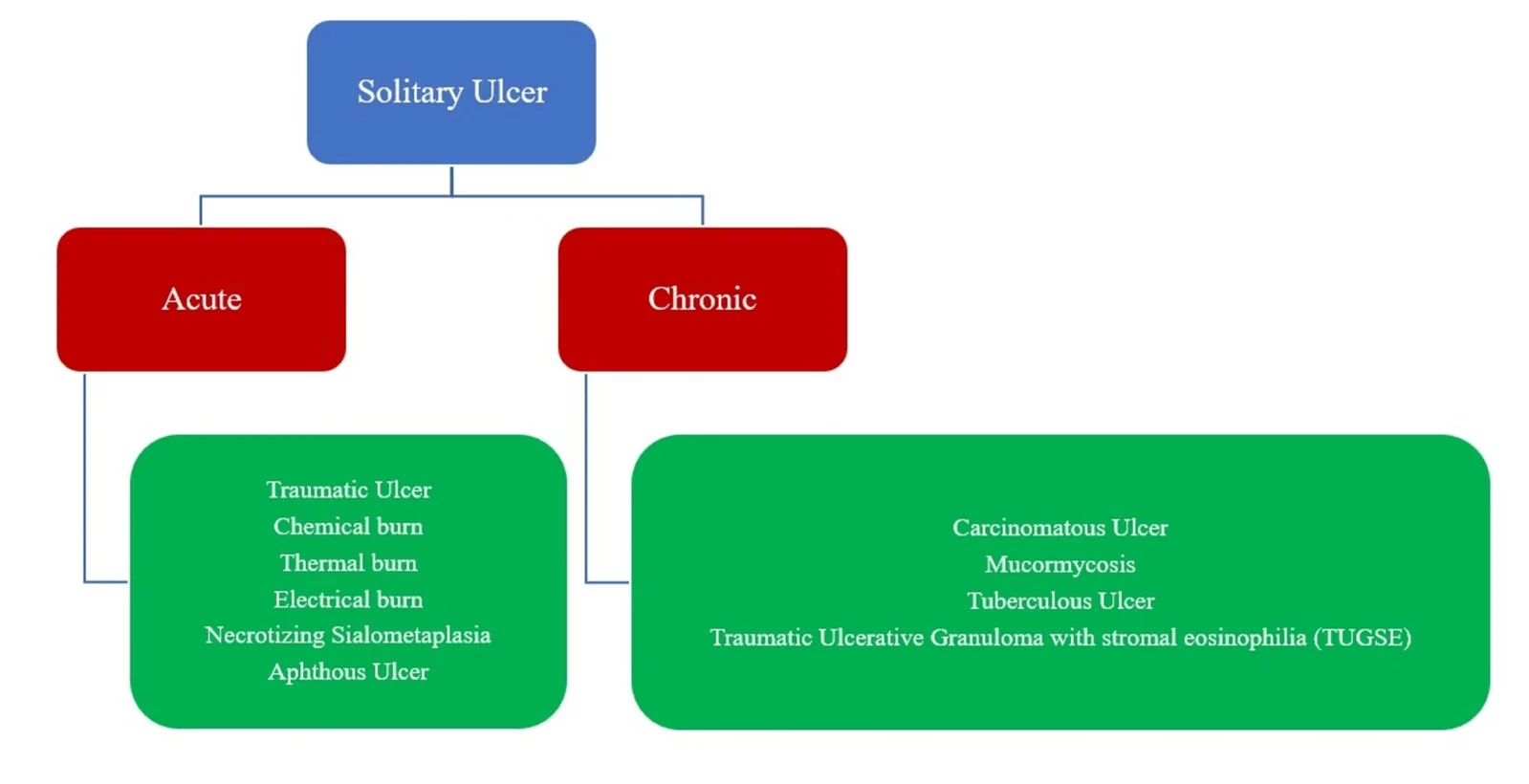
Classification of solitary ulcers Image Credit: Author

TUGSE is a chronic self-limiting inflammation that extends into the underlying structure. Microscopically, it shows an eosinophilic and lymphocytic inflammatory cell infiltrate extending to the underlying skeletal muscle. Other conditions, such as squamous cell carcinoma and deep-seated fungal infections, may mimic the clinical presentation of chronic traumatic ulcers, particularly TUGSE [4].

Therefore, a biopsy is recommended for all oral ulcers that prolong for more than two weeks despite removing any identifiable local irritation factor, such as a sharp tooth cusp. It is impossible to identify the cause of the traumatic ulcer in some cases, and, in rare cases, it may be factual. Traumatic ulcers are often covered by sharply demarcated borders. Most traumatic ulcers will be resolved and should remain observed until they are healed. In chronic lesions, ulceration is commonly accompanied by a white border of hyperkeratosis [5]. It helps speed up healing and decrease pain by applying topically applied corticosteroids such as clobetasol propionate 0.05% gel. For TUGSE, intralesional corticosteroid injections could be beneficial [6].

Extremely prevalent, the traumatic oral ulcer can be caused by acute or chronic trauma by sharp objects, accidental or intentional biting, or excessive brushing. The ulcer is often single and can range in diameter from less than 1 mm to greater than 1 cm. During the first several days following a traumatic incident, the patient typically has minor pain. With the eradication of the underlying cause, recovery occurs within two to seven days. A superimposed candidal infection can delay the healing process even in healthy people. The tongue, lips, and buccal mucosa are common sites of damage caused by teeth. Ulcers on the gingiva, palate, and mucobuccal fold are typically caused by food with hard, jagged edges or toothbrushes (induced trauma). A thorough patient interview and clinical examination aid in diagnosing an ulcer [7].

Ulcers caused by mucormycosis usually have a black eschar and clinically exposed underlying alveolar bone [8]. The self-inflicted accidental injury to the lip is called the Morsicatio labiorum [9]. Necrotizing sialometaplasia occurs more commonly on the posterior hard palate and near the junction of the hard and soft palate [10]. The differential traumatic ulcer diagnoses include eosinophilic ulcer (TUGSE) and carcinomatous ulcer. In carcinomatous ulcers, the base is indurated and tender [10]. Head and neck tuberculosis (HNT) also occurs in the oral cavity in about 5%of cases in addition to cervical lymph nodes (71.67%) [11]. Doxycycline is helpful in the treatment of recurrent aphthous ulcers and herpes labialis [12]. Aloe vera gel (80%) effectively prevents traumatic ulcers caused by fixed orthodontic appliances [13]. Erbium, chromium-doped yttrium, scandium, gallium and garnet (Er,Cr:YSGG) laser effectively promotes healing and relieves the pain in traumatic ulcers [14].

## Conclusions

The diagnosis of an ulcer remains challenging to the oral physician. The clinician must not overlook the occurrence of an ulcer in the oral cavity. Careful examination includes a detailed, complete history taking, mode of onset, solitary or multiple, which helps narrow the multifactorial causes of oral ulcers. The exact causes must be identified to prevent unwanted invasive surgical procedures. The etiological factors of ulcers must be explained to the patient to prevent apprehension and anxiety.

## References

[1] Fitzpatrick SG, Cohen DM, Clark AN (2019). Ulcerated lesions of the oral mucosa: clinical and histologic review. Head Neck Pathol.

[2] Gilvetti C, Porter SR, Fedele S (2010). Traumatic chemical oral ulceration: a case report and review of the literature. Br Dent J.

[3] Goyal R, Jadia S, Jain L, Agarawal C (2016). A clinical study of oral mucosal lesions in patients visiting a tertiary care centre in central India. Indian J Otolaryngol Head Neck Surg.

[4] Aizic A, Raiser V, Solar I, Aharon Z, Shlomi B, Kaplan I (2019). Traumatic ulcerative granuloma with stromal eosinophilia: CD30 analysis and clonality for T cell receptor gene re-arrangement. Acta Histochem.

[5] Hamie L, Hamie M, Kurban M, Abbas O (2022). Eosinophilic ulcer of the oral mucosa: an update on clinicopathologic features, pathogenesis, and management. Int J Dermatol.

[6] Philipone EM, Peters SM (2023). Ulcerative and inflammatory lesions of the oral mucosa. Oral Maxillofac Surg Clin North Am.

[7] Lourenço SV, Silva MA, Nico MM (2005). An ulcer on the lip. Clin Exp Dermatol.

[8] Mora-Martínez A, Murcia L, Rodríguez-Lozano FJ (2023). Oral manifestations of mucormycosis: a systematic review. J Fungi (Basel).

[9] Kang HS, Lee HE, Ro YS, Lee CW (2012). Three cases of 'morsicatio labiorum'. Ann Dermatol.

[10] Gupta A, Priyadarshinee A, Rao LN, Redhu A, Jha KS (2021). Necrotizing sialometaplasia-a self-limiting condition which poses a diagnostic dilemma. Clin Case Rep.

[11] Pang P, Duan W, Liu S (2018). Clinical study of tuberculosis in the head and neck region-11 years' experience and a review of the literature. Emerg Microbes Infect.

[12] Piacentini M, Borghetti RL, Zancanaro de Figueiredo MA, Cherubini K, Gonçalves Salum F (2019). Doxycycline: an option in the treatment of ulcerated oral lesions?. J Clin Pharm Ther.

[13] Leiva-Cala C, Lorenzo-Pouso AI, Centenera-Centenera B, López-Palafox J, Gándara-Vila P, García-García A, Pérez-Sayáns M (2020). Clinical efficacy of an Aloe Vera gel versus a 0.12% chlorhexidine gel in preventing traumatic ulcers in patients with fixed orthodontic appliances: a double-blind randomized clinical trial. Odontology.

[14] Kurtulmus-Yilmaz S, Yilmaz HG, Tumer H, Sadettinoglu K (2015). Clinical evaluation of the Er,Cr:YSGG Laser therapy in the treatment of denture-related traumatic ulcerations: a randomized controlled clinical study. Photomed Laser Surg.

